# Supplementary material for: Dynamic changes in neuronal and glial GAL4 driver expression during Drosophila aging
Source: Genetics. 2025 Feb 14;229(3):iyaf014. doi: 10.1093/genetics/iyaf014 (PMC11912842; doi:10.1093/genetics/iyaf014)
Supplement: iyaf014_Supplementary_Data [file iyaf014_supplementary_data.pdf]

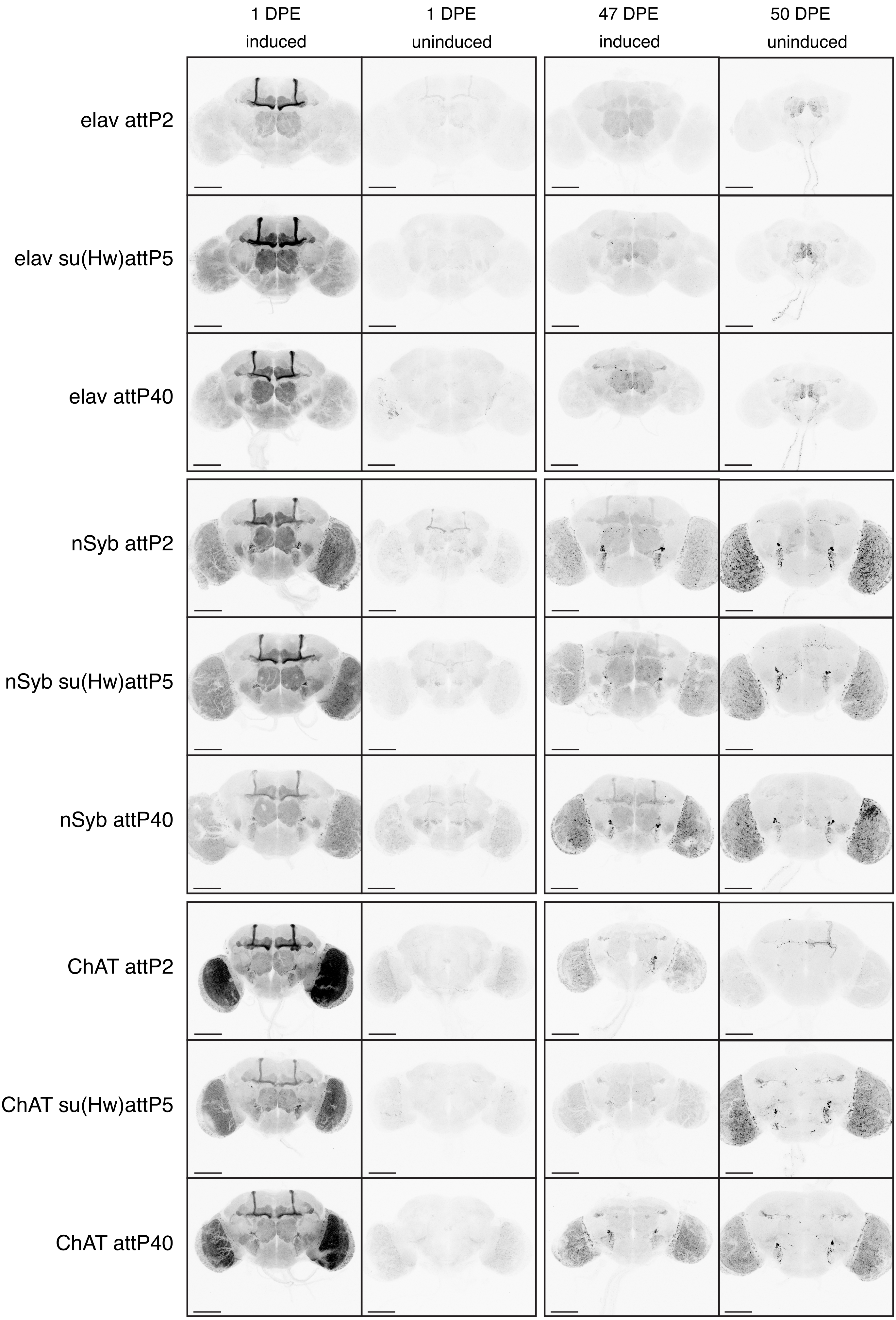

Figure S1. Comparison between brains from flies that were induced at 29°C for 24 hours at 1 or 47 DPE and flies that were left uninduced at 18°C until 1 or 50 DPE. Scale bar, 100 μm.

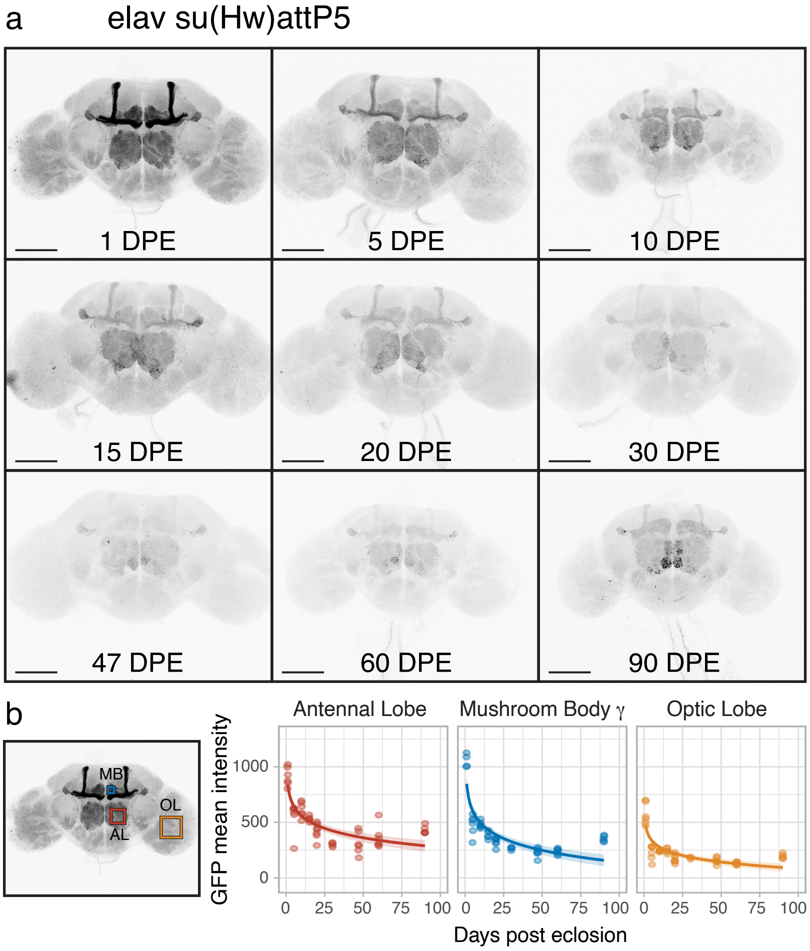

Figure S2. (a) Flies expressing membrane-bound GFP (inserted in su(Hw)attP5) under the control of *elav[C155]-GAL4* and a temperature-sensitive GAL80 were induced for 24-hour time windows by shifting from 18°C to 29°C at nine time points throughout adult life (up to 90 DPE). (b) Three brain regions were chosen for quantification of GFP mean intensity. Logarithmic regression models were fitted and summary statistics can be found in Table S1. Scale bar, 100 µm.

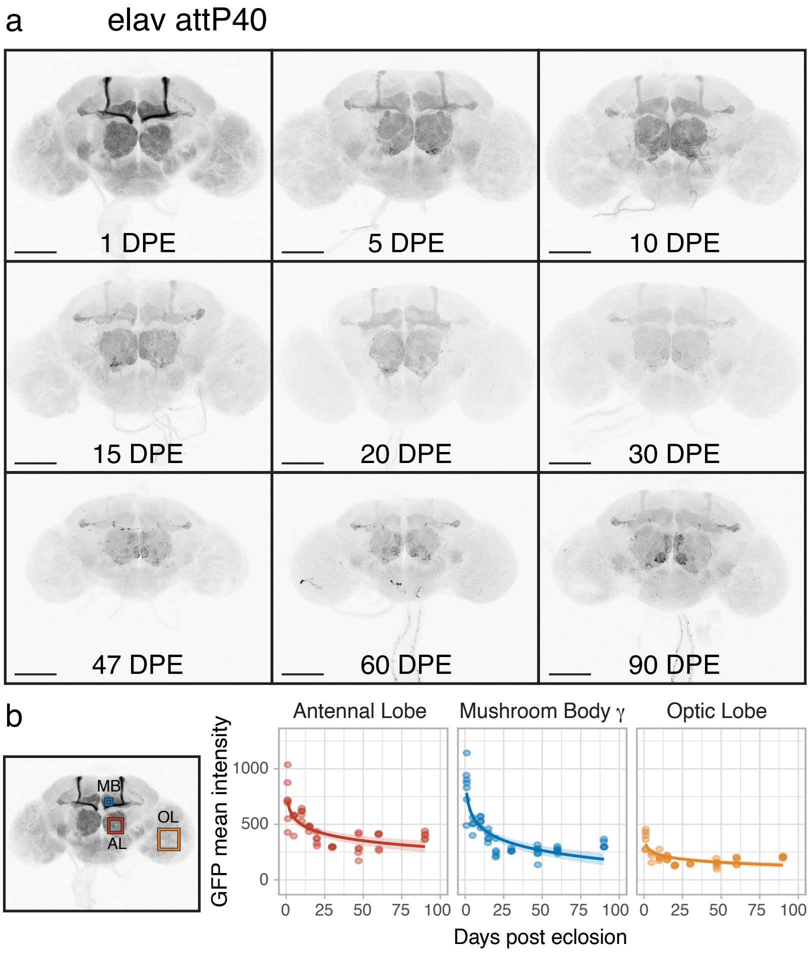

Figure S3. (a) Flies expressing membrane-bound GFP (inserted in attP40) under the control of *elav[C155]-GAL4* and a temperature-sensitive GAL80 were induced for 24-hour time windows by shifting from 18°C to 29°C at nine time points throughout adult life (up to 90 DPE). (a) Three brain regions were chosen for quantification of GFP mean intensity. Logarithmic regression models were fitted and summary statistics can be found in Table S1. Scale bar, 100 µm.

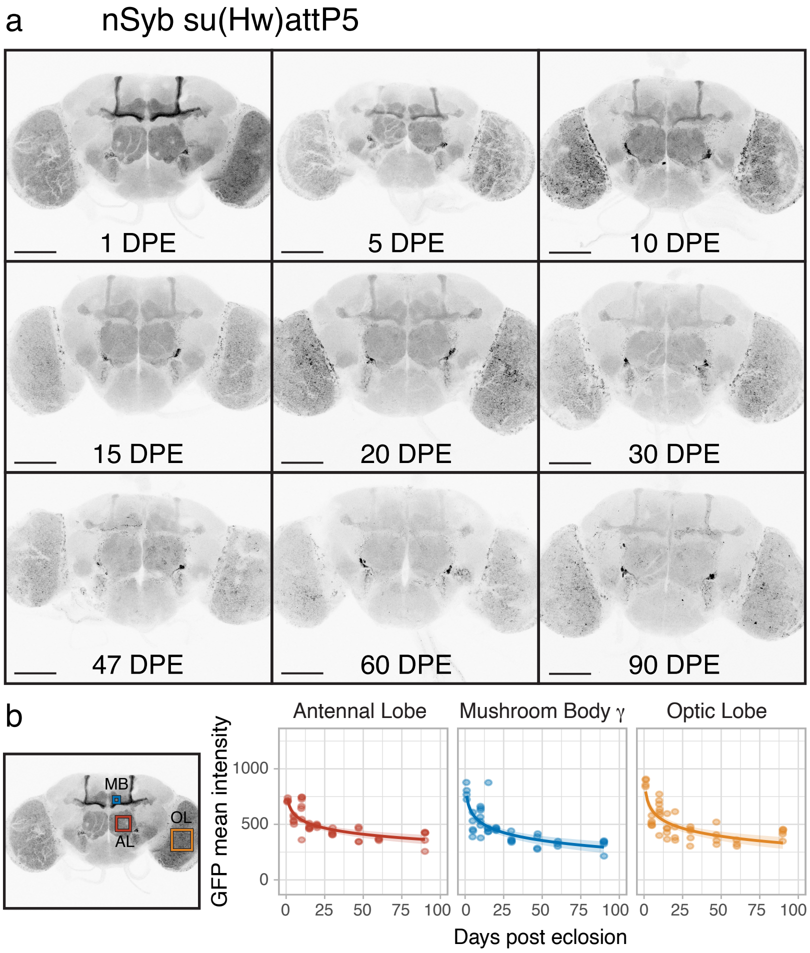

Figure S4. (a) Flies expressing membrane-bound GFP (inserted in su(Hw)attP5) under the control of *nSyb[R57C10]-GAL4* and a temperature-sensitive GAL80 were induced for 24-hour time windows by shifting from 18°C to 29°C at nine time points throughout adult life (up to 90 DPE). (b) Three brain regions were chosen for quantification of GFP mean intensity. Logarithmic regression models were fitted and summary statistics can be found in Table S1. Scale bar, 100 μm.

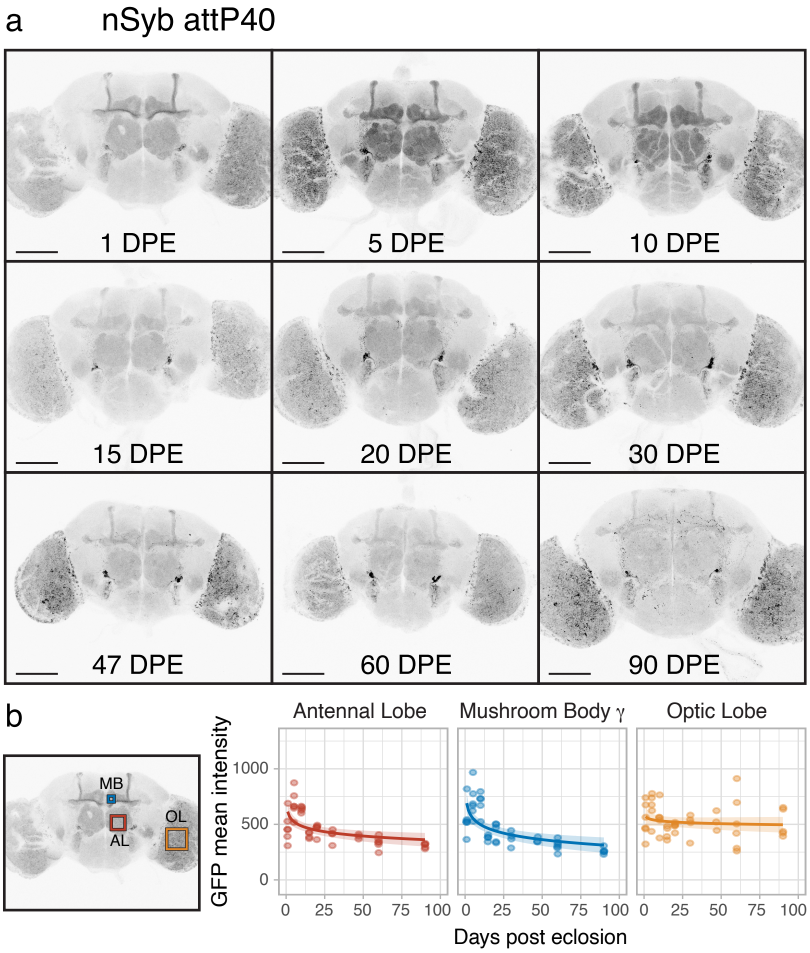

Figure S5. (a) Flies expressing membrane-bound GFP (inserted in attP40) under the control of *nSyb[R57C10]-GAL4* and a temperature-sensitive GAL80 were induced for 24-hour time windows by shifting from 18°C to 29°C at nine time points throughout adult life (up to 90 DPE). (b) Three brain regions were chosen for quantification of GFP mean intensity. Logarithmic regression models were fitted and summary statistics can be found in Table S1. Scale bar, 100 µm.

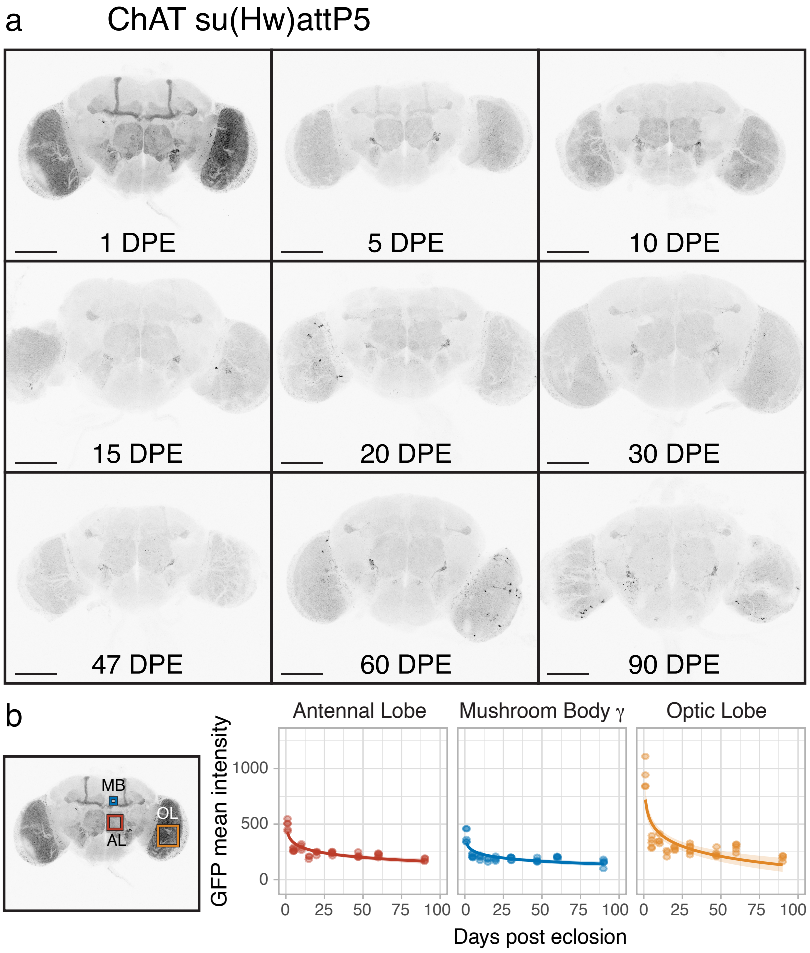

Figure S6. (a) Flies expressing membrane-bound GFP (inserted in su(Hw)attP5) under the control of *ChAT-GAL4* and a temperature-sensitive GAL80 were induced for 24-hour time windows by shifting from 18°C to 29°C at nine time points throughout adult life (up to 90 DPE). (b) Three brain regions were chosen for quantification of GFP mean intensity. Logarithmic regression models were fitted and summary statistics can be found in Table S1. Scale bar, 100 μm.

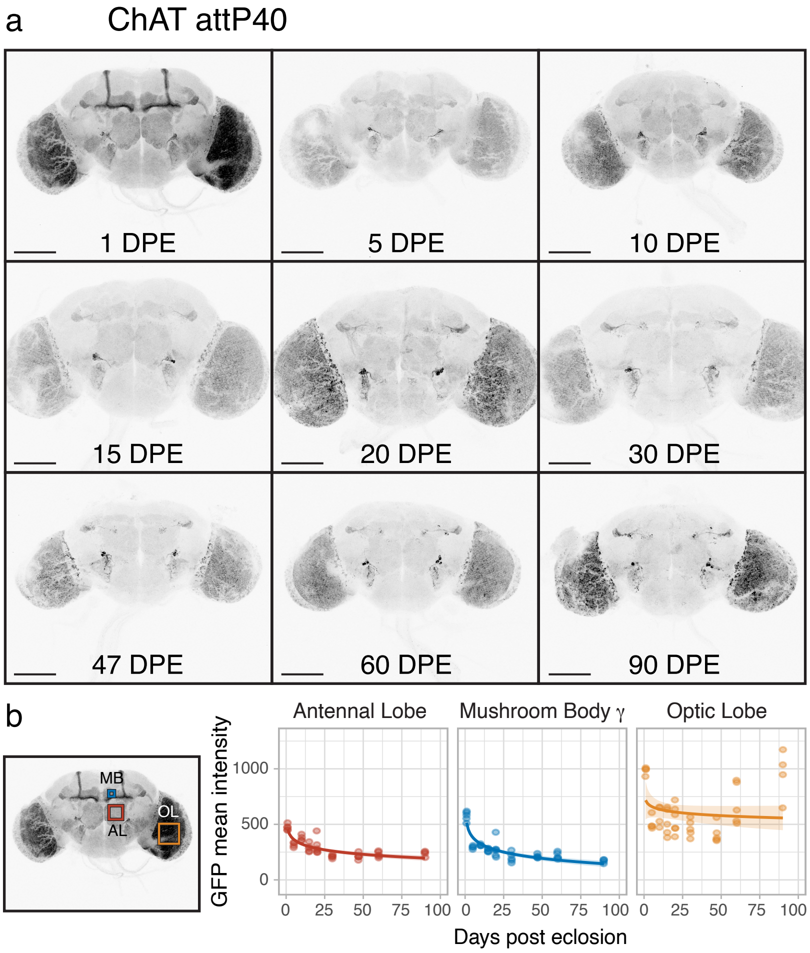

Figure S7. (a) Flies expressing membrane-bound GFP (inserted in attP40) under the control of *ChAT-GAL4* and a temperature-sensitive GAL80 were induced for 24-hour time windows by shifting from 18°C to 29°C at nine time points throughout adult life (up to 90 DPE). (b) Three brain regions were chosen for quantification of GFP mean intensity. Logarithmic regression models were fitted and summary statistics can be found in Table S1. Scale bar, 100 µm.

attP site	Brain Region	GAL4	Intercept Estimate	Intercept Std Error	Intercept CI Lower	Intercept CI Upper	Intercept Adjusted p-value	Log(DPE) Estimate	Log(DPE) Std Error	Log(DPE) CI Lower	Log(DPE) CI Upper	Log(DPE) Adjusted p-value
attP2	Antennal Lobe	ChAT	464.54	21.07	422.11	506.98	< 2E-16	-59.86	6.93	-73.81	-45.90	1.03E-10
attP2	Optic Lobe	ChAT	1061.11	81.37	896.65	1225.57	6.54E-16	-165.11	26.43	-218.53	-111.69	2.78E-07
attP2	Mushroom Body gamma	ChAT	604.05	27.77	548.11	659.98	< 2E-16	-110.58	9.13	-128.98	-92.19	9.39E-15
attP2	Mushroom Body alpha 3	ChAT	774.08	73.98	625.07	923.09	1.32E-13	-188.85	24.33	-237.86	-139.84	1.28E-09
attP40	Antennal Lobe	ChAT	456.62	17.31	421.66	491.59	< 2E-16	-57.92	5.55	-69.12	-46.71	1.36E-12
attP40	Optic Lobe	ChAT	813.74	87.28	637.48	990.01	1.10E-11	-63.56	27.97	-120.04	-7.09	0.029313
attP40	Mushroom Body gamma	ChAT	514.08	20.06	473.56	554.60	< 2E-16	-81.96	6.43	-94.95	-68.98	9.39E-15
su(Hw)attP5	Antennal Lobe	ChAT	426.64	16.37	393.55	459.73	< 2E-16	-57.92	5.29	-68.60	-47.24	5.55E-13
su(Hw)attP5	Optic Lobe	ChAT	720.64	47.34	624.80	816.47	< 2E-16	-130.66	14.96	-160.95	-100.37	2.96E-10
su(Hw)attP5	Mushroom Body gamma	ChAT	331.31	15.66	299.67	362.94	< 2E-16	-42.40	4.99	-52.48	-32.31	3.04E-10
attP2	Antennal Lobe	elav	509.85	33.14	442.97	576.72	< 2E-16	-59.68	10.69	-81.26	-38.10	1.98E-06
attP2	Optic Lobe	elav	176.74	12.33	151.82	201.66	< 2E-16	-17.88	4.00	-25.97	-9.79	7.33E-05
attP2	Mushroom Body gamma	elav	687.02	36.82	612.71	761.32	< 2E-16	-118.73	11.88	-142.70	-94.75	3.44E-12
attP2	Mushroom Body alpha 3	elav	696.52	67.97	559.34	833.70	5.58E-13	-152.97	21.94	-197.24	-108.70	2.26E-08
attP40	Antennal Lobe	elav	706.84	36.05	634.19	779.50	< 2E-16	-90.54	11.67	-114.07	-67.01	1.36E-09
attP40	Optic Lobe	elav	322.47	19.21	283.63	361.30	< 2E-16	-42.03	6.11	-54.38	-29.69	3.74E-08
attP40	Mushroom Body gamma	elav	784.10	36.80	709.94	858.26	< 2E-16	-132.93	11.92	-156.95	-108.92	1.23E-13
su(Hw)attP5	Antennal Lobe	elav	841.40	36.42	768.21	914.58	< 2E-16	-122.77	11.82	-146.53	-99.01	2.85E-13
su(Hw)attP5	Optic Lobe	elav	471.65	25.85	419.64	523.65	< 2E-16	-84.10	8.37	-100.95	-67.26	1.04E-12
su(Hw)attP5	Mushroom Body gamma	elav	960.78	47.93	864.46	1057.10	< 2E-16	-186.42	15.56	-217.69	-155.15	9.39E-15
attP2	Antennal Lobe	nSyb	746.20	30.52	684.61	807.79	< 2E-16	-92.31	9.71	-111.89	-72.72	1.34E-11
attP2	Optic Lobe	nSyb	736.36	59.07	617.05	855.66	1.81E-15	-44.98	18.94	-83.23	-6.73	0.023904
attP2	Mushroom Body gamma	nSyb	841.00	46.29	747.58	934.42	< 2E-16	-115.91	14.72	-145.62	-86.21	1.33E-09
attP2	Mushroom Body alpha 3	nSyb	936.34	47.65	840.17	1032.50	< 2E-16	-185.23	15.15	-215.81	-154.64	1.52E-14
attP40	Antennal Lobe	nSyb	614.47	40.73	532.28	696.66	< 2E-16	-56.17	13.36	-83.13	-29.20	1.49E-04
attP40	Optic Lobe	nSyb	569.07	47.68	472.78	665.35	7.08E-15	-16.13	15.51	-47.46	15.19	0.304447
attP40	Mushroom Body gamma	nSyb	690.60	46.15	597.47	783.73	< 2E-16	-83.91	15.14	-114.46	-53.36	2.16E-06
su(Hw)attP5	Antennal Lobe	nSyb	724.98	29.86	664.62	785.34	< 2E-16	-80.27	9.78	-100.04	-60.49	7.41E-10
su(Hw)attP5	Optic Lobe	nSyb	796.09	37.86	719.56	872.62	< 2E-16	-103.03	12.41	-128.11	-77.96	5.74E-10
su(Hw)attP5	Mushroom Body gamma	nSyb	755.72	36.89	681.16	830.28	< 2E-16	-102.56	12.09	-126.98	-78.13	3.52E-10

Table S1. Summary statistics for the logarithmic regression model fitted to the data for all neuronal drivers used in this study. The Benjamini-Hochberg method was used to adjust P values for multiple testing.
